# Neoadjuvant treatment of esophageal cancer: chemotherapy, chemoradiation, immunotherapy, and future trends of therapy

**DOI:** 10.1515/iss-2023-0005

**Published:** 2024-11-25

**Authors:** Freschta Malekzada, Miljana Vladimiriov, Michael Leitz, Julia Michel, Fabian Nimzewski, Jens Hoeppner

**Affiliations:** Department of Surgery, University Medical Center Schleswig-Holstein, Campus Lübeck, Lübeck, Germany; Department of Surgery, Bielefeld University, Medical School and University Medical Center OWL, Klinikum Lippe, Detmold, Germany

**Keywords:** esophageal cancer, chemotherapy, chemoradiation, immunotherapy, multimodal treatment

## Abstract

In the Western hemisphere, nonmetastatic locally advanced esophageal carcinoma is mostly treated in multimodal therapy protocols according to current therapy guidelines. In squamous cell carcinoma of the esophagus, neoadjuvant chemoradiation is the favorable option. Unimodal surgical and chemoradiation treatment alternatives show inferior results on this entity. For locally advanced adenocarcinoma of the esophagus perioperative chemotherapy and neoadjuvant chemoradiation have been competing treatment approaches in the recent past. Both are evidence based (class I evidence) and superior compared to unimodal surgery. However, the latest results of head-to-head comparative therapy studies show superior overall survival results for the FLOT regimen of perioperative chemotherapy. Furthermore, immunotherapy and targeted therapy with monoclonal antibodies have become a strong focus of current clinical research. Nivolumab as well as trastuzumab are already an important part of the current standard therapies. In both entities – SCC and AC – a significant quota of patients shows a locoregional complete remission of the tumor in the specimen after modern neoadjuvant therapy and surgical resection. The addition of immunotherapy and targeted therapy to neoadjuvant therapy further increases complete remission rates in defined subgroups according to the results of current studies. Currently, three prospective randomized trials are ongoing on the subject of future possibilities for organ-preserving concepts in case of complete clinical remission (“surgery as needed,” “watch and wait”). It is to be expected for the future that curative short-term and long-term treatment results in locally advanced esophageal carcinoma will significantly improve, particularly due to the additional possibilities of immunotherapy and organ-preserving therapy concepts in postneoadjuvant complete remission.

## Introduction

Esophageal cancer is ranked seventh most common tumor worldwide and sixth in terms of overall mortality according to the Global Cancer Statistics 2018 [1]. The 5-year survival was last estimated between 10 and 30 % depending on tumor stage [2]. Therapeutic options for nonmetastatic disease have evolved from sole radical surgical resection of the esophagus alone to multimodality therapy through innovation in the development of more minimally invasive surgical technique and advanced drug therapies [3]. Concurrently the surgical approach has changed toward minimally invasive access techniques, with retention of radical esophageal organ resection and systematic locoregional lymphadenectomy [4]. However, therapy is not the same for every esophageal cancer and should be personalized for every patient according to current Western recommendations [5], [6], [7]. For the curative therapeutic approach, endoscopic, surgical, or multimodality therapy can be considered depending on histologic subtype and staging according to the TNM classification. The guidelines of the National Comprehensive Cancer Network in the United States, the evidence-based guidelines of the American society of clinical oncology and the German guidelines list multimodal therapy options [5], [6], [7].

Multimodal therapy as a standard treatment is particularly recommended for locally advanced, nonmetastatic esophageal cancer [5], [6], [7]. Neoadjuvant medical and radiation therapy, surgery, and adjuvant medical therapy are the components of multimodal treatment of esophageal cancer.

Neoadjuvant therapy is given before surgery and the benefits of neoadjuvant treatment include the following:

– Downstaging of the tumor: Neoadjuvant treatment can shrink the tumor size, making it easier to remove surgically, and increasing the chance of cure.
– Improved surgical outcomes: Neoadjuvant treatment can make the tumor smaller, less aggressive and make it easier to remove, which can lead to fewer complications and better surgical outcomes.
– Reduced risk of positive surgical margins: Neoadjuvant treatment can decrease the risk of cancer cells remaining at the edges of the tissue removed during surgery, which is called positive surgical margins.
– Better control of symptoms: Neoadjuvant treatment can help to control symptoms such as pain, difficulty swallowing, and weight loss.

Adjuvant treatment for esophageal cancer refers to treatment given after the main treatment by surgery in order to reduce the risk of the cancer returning. The benefit of adjuvant treatment is that it can increase the chances of a cure and prolong survival for patients with esophageal cancer.

In this review article, neoadjuvant chemotherapy or chemoradiation of adenocarcinoma (AC) and squamous cell carcinoma (SCC) is described in consensus with current guidelines and recent advances in the study of complementary therapies and future development of multimodal therapeutic algorithms are sketched. Specifically, the use of immunotherapeutic drugs has come into scientific focus, with current studies showing promising results in combination with neoadjuvant chemoradiation [8].

## Squamous cell carcinoma

**Figure 1:**
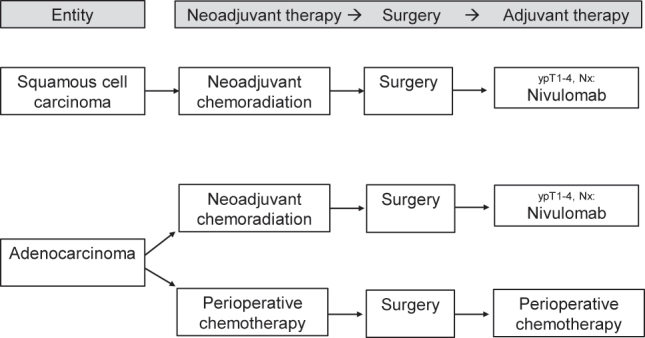
Current multimodal algorithm for the treatment of locally advanced esophageal cancer.

For locally advanced SCC of the esophagus, neoadjuvant chemoradiation followed by esophagectomy with systematic lymphadenectomy is the current western standard of treatment (Figure 1). In SCC, a high sensitivity of the tumors to neoadjuvant chemoradiation has been proved: The Dutch CROSS study (41.4 Gy and carboplatin/paclitaxel prior to esophagectomy) showed a complete tumor remission rate of 49 % in SCC compared to 23 % in AC [9]. The prospective randomized comparison was done with unimodal esophagectomy *versus* neoadjuvant chemoradiation plus esophagectomy in the experimental arm. Especially in the subgroup of SCC, a significant survival benefit for the multimodally treated patients has been proved (overall survival 82 vs. 21 months; hazard ratio [HR] 0.422; 95 % confidence interval [CI] 0.226–0.788; [9]). Although only a small subgroup of the patients with SCC was treated with neoadjuvant chemoradiation plus surgery in the trial (n=41), the protocol with 41.4 Gy plus carboplatin/paclitaxel has become a western standard protocol for multimodal treatment of nonmetastatic SCC. Further chemotherapeutic components used in clinical routine of chemoradiation are cisplatin/5-fluorouracil (5-FU) or FOLFOX (folinic acid, 5-FU, oxaliplatin) [10], 11]. The volume of the neoadjuvant radiation dose is still under debate. Currently, mostly radiotherapeutic doses between 41.4 and 50.4 Gy are used in the neoadjuvant indication. Retrospective analyses of US National Cancer Database compared low and high neoadjuvant radiation doses (41.4 vs. 50.4 Gy) and showed reduced 90-day perioperative mortality when using lower dosages (2.3 vs. 6.5 %; p=0.01). Moreover, significant advantages in overall survival (median overall survival 53 vs. 41 months; p=0.01) in the low-dose group, comparable R0 rates (93.2 vs. 92.4 %; p=0.678) and comparable postneoadjuvant complete remission rates (19.3 vs. 21.5 %; p=0.442) were evident in the study [12], 13] (Figure 1).

In comorbid patients, definitive chemoradiation is a valuable option for curative treatment: Two small prospective randomized studies have shown no difference in overall survival comparing definitive and neoadjuvant chemoradiation [14], 15]. In 2016, a Cochrane review found equivalent results in survival when comparing definitive chemoradiation to esophagectomy on the basis of studies with an overall worse quality of comparative data and a very high data heterogeneity [16]. Comparing definitive chemoradiation neoadjuvant chemoradiation plus esophagectomy, a current registry analysis from the National Cancer Database in the United States provided different results: A retrospective propensity score analysis comparing 1816 patients after definitive chemoradiation to 1861 patients after neoadjuvant chemoradiation plus esophagectomy provided a significant survival benefit for the multimodally treated patients (overall survival 18 vs. 36.5 months; HR 0.58; 95 % CI 0.53–0.63; p<0.001) [17].

## Adenocarcinoma

In locally advanced AC of the esophagus and the gastroesophageal junction, neoadjuvant chemoradiation with 41.4 Gy plus carboplatin/paclitaxel improves the median survival compared to surgery alone in the prospective randomized comparison (43 vs. 27 months; HR 0.73; 95 % CI 0.524–0.998) [9]. This effect is less pronounced than in SCC and seen on its own in the AC subgroup not even significant, but still forms the base of evidence for a wide international application of neoadjuvant chemoradiation in AC.

In AC, not only neoadjuvant chemoradiation but also perioperative chemotherapy without radiotherapy improves oncologic outcomes compared to surgery alone according to level 1 evidence. Compared to surgery alone, perioperative chemotherapeutic protocols with epirubricin/cisplatin/5-FU (5-year survival 36 vs. 23 %; HR 0.75) and cisplatin/5-FU (5-year survival 38 vs. 24 %; HR 0.69) proved significant survival benefits in two prospectively randomized trials [18], 19]. Both trials have been carried out in mixed collectives containing patients with gastric cancer and esophageal AC.

The current chemotherapeutic standard in AC, however, is the perioperative FLOT therapy (5-FU, leukovorin, oxaliplatin, docetaxel) with a median overall survival defined of 50 months [20]. A prospective randomized comparison between FLOT plus surgery vs. ECF (epirubicin, cisplatin, fluorouracil)/ECF/EOX (epirubicin, oxaliplatin, capecitabine) plus surgery in 716 patients proved a significant survival benefit for the patients treated in the FLOT arm (median survival 50 vs. 35 month; HR 0.77) [20].

Which of the two competing multimodal therapy concepts – neoadjuvant chemoradiation or perioperative chemotherapy – is to be given preference with regard to short- and long-term results in esophageal and junctional AC has been unclear for years and was internationally disputed [21]. The question is the subject of multicentric prospective randomized ESOPEC trial [22]. The ESOPEC trial compares neoadjuvant chemoradiation (CROSS protocol: 41.4 Gy, carboplatin/paclitaxel) vs. perioperative chemotherapy (FLOT protocol: 5-FU, leukovorin, oxaliplatin, docetaxel) in a collective of patients with exclusively esophageal and junctional AC. The trial has published its first results recently and found superior overall survival results for the FLOT regimen of perioperative chemotherapy compared to CROSS chemoradiation. The 3-year OS rates in ESOPEC were 57.4 % (95 % CI 50.1–64.0 %) for FLOT and 50.7 % (95 % CI 43.5–57.5 %) for CROSS (HR 0.70, 95 % CI 0.53–0.92, p=0.012) [23]. It is expected that these results will influence national and international treatment guidelines in terms of recommending perioperative chemotherapy for locally advanced esophageal adenocarcinoma (Figure 1). In the same context, the ongoing German RACE trial compares the classic perioperative FLOT regime with an experimental regime of neoadjuvant FLOT plus chemoradiation in the study arm [24].

As an important aspect for the surgeon, both multimodal regimens – neoadjuvant chemoradiation and perioperative chemotherapy – do not impair perioperative morbidity and mortality compared to unimodal surgical regimens [9], 18], 19]. Postoperative mortality for the current multimodal protocols is 4–8 % according to the major prospective clinical trials [9], [18], [19], [20].

## Immunotherapy and targeted therapy in of perioperative anticancer therapy

In the last decade, immunotherapeutic drugs and biologicals have been introduced to standard multimodal and perioperative treatment algorithms of nonmetastatic esophageal cancer (Table 1). The common mechanism of action of these drugs is the binding of the drug to a receptor or specific ligand resulting in interruption of intracellular signal for tumor progression (Table 1).

**Table 1:** Immunotherapeutics/biologicals in perioperative therapy of esophageal cancer and gastroesophageal junction for adenocarcinoma (AC) and squamous cell carcinoma (SCC).

Immunotherapeutics/biologicals: Current studies						
Name	Checkmate 577	Keynote 585	Infinity	Dante	PETRARCA	EORTC-1203
Type of study	Phase-III	Phase-III	Phase-II	Phase-II	Phase-II	Phase-III
Study drug	Nivolumab	Pembrolizumab	Tremellmumab Durvalumab	Atezolizumab	Trastuzumab Pertuzumab	Trastuzumab
Receptor/Ligand	PD-1	PD-1	CTLA 4 PD-L1	PD-L1	HER2	HER2
Histology	AC/SCC	AC	AC	AC	AC	AC
Reference	8	29	30	28	26	27

One target receptor on the tumor cell is the human epidermal growth factor receptor Type 2 (HER2). In the metastatic situation, the HER2 antibody trastuzumab has been established for more than a decade and has been proven to be effective in HER2-positive adenocarcinomas [25]. In the locally advanced HER2-positive curative situation, German PETRARCA trial examined the combination FLOT ± trastuzumab and pertuzumab vs. FLOT alone in a comparative prospectively randomized trial design. The trial showed an almost three times increased rate of histopathological complete responders (35 vs. 12 %) after FLOT plus antibody compared to perioperative FLOT therapy alone [26]. The same question is currently being asked in perioperative settings for trastuzumab with and without combination with pertuzumab in the ongoing EORTC-1203 trial in HER2-positive junctional and gastric adenocarcinoma [27].

Another effective target in immunotherapy of esophageal cancer is the PD-1 receptor and its ligand PD-L1 by the so-called checkpoint inhibitors.

Checkpoint inhibitors are a type of immunotherapy that work by blocking specific proteins on cancer cells that help them evade the immune system. These proteins, known as checkpoint molecules, act as “brakes” on the immune system, preventing it from recognizing and attacking cancer cells. Checkpoint inhibitors are a type of monoclonal antibody designed to target specific checkpoint molecules, such as PD-1 and CTLA-4, which are found on the surface of cancer cells. By blocking these molecules, checkpoint inhibitors release the “brakes” on the immune system and allow it to recognize and attack cancer cells. This leads to an increase in the number of immune cells that specifically target cancer cells and a reduction in the growth and spread of the cancer.

The activation of PD-1 expressed on T cells results in an inhibitory effect on the immune response of these cells. Checkpoint inhibitors block this effect and “reactivate” the immune response against PD-L1 expressing on tumor cells. Very promising results have recently been obtained for adjuvant treatment with the PD-1 antibody nivolumab in the prospectively randomized Checkmate 577 trial for the nonmetastatic SCC and AC after neoadjuvant chemoradiation and esophagectomy [8]. The adjuvant administration of nivolumab in patients with residual disease in the operative specimen for an average of 10 months improved disease-free survival significantly from 11 months in the placebo group to 22 months in the verum group (HR 0.69; 96.4 % CI 0.56–0.86; p<0.001). The study demonstrated this effect for AC (HR 0.75; 95 % CI 0.56–0.96) and even more pronounced for SCC (HR 0.61; 95 % CI 0.42–0.88) [8].

In contrast, the addition of the PD1 antibody pembrolizumab to perioperative chemotherapy was not able to improve survival outcomes in locally advanced gastric and esophagogastric cancers in the prospective randomized Keynote 585 trial [28].

Against this background, immunotherapy combined with chemoradiation currently appears to be able to have an additional effect on survival. The combination of immunotherapy with perioperative chemotherapy, on the other hand, does not appear to have an additional improving effect on patient survival according to current studies.

One explanation for this could be that immunotherapy has no or a smaller additional systemic effect in the overall more systemically effective protocols of perioperative chemotherapy compared to the less systemically effective protocols of neoadjuvant radiochemotherapy.

Currently, the PD-L1 inhibitor atezolizumab is examined in the neoadjuvant setting in the perioperative treatment of gastric and junctional AC in combination with FLOT in the DANTE trial (EudraCT: 2017-001979-23). Interim results show a clear increase of postneoadjuvant complete remission rates from 15 to 27 % in the overall collective and from 27 to 50 % in MSI high patients [29].

Other studied checkpoint inhibitors for perioperative therapy protocols in esophageal carcinoma tested in ongoing comparative therapy studies are tremelimumab and durvalumab [30].

## Clinical complete remission: active surveillance or obligate esophagectomy

The neoadjuvant therapy component in the multimodal treatment protocols is characterized by an increasing antitumor effectiveness. Especially the view of the postneoadjuvant complete remission rate of meanwhile 20–30 % after preoperative chemotherapy or chemoradiation is underlining the question of dependable clinical identification of tumor complete remission and systematic clinical surveillance of complete remission. Proposed future treatment algorithms will possibly execute esophagectomy only in the case of postneoadjuvant tumor residue or tumor recurrence but not as an obligate part of treatment.

A recent scoping review described all relevant clinical studies and collected the comparative evidence from studies comparing direct postneoadjuvant surgery vs. postneoadjuvant surveillance and surgery as needed. Three completed randomized controlled trials (RCTs) including 468 participants, three planned/ongoing RCTs with a planned sample size of 752 participants, one nonrandomized controlled study with 53 participants, 10 retrospective cohort studies (2,228 participants), and one survey on patients’ preferences (100 participants) were identified. The scoping review revealed that although surveillance and surgery as needed has been investigated within different study designs, the available study pool showed severe methodological limitations, and clinical results were very heterogeneous [31]. All further evidence regarding this question is indirect and based on results from interventions and observational studies as well as on results from trials on multimodal treatment of esophageal carcinoma neoadjuvant chemotherapy or neoadjuvant chemoradiation. These data show pathological complete remission rates in AC in overall 16–30 % of the patients after neoadjuvant chemotherapy with FLOT, with notably higher pathological complete remission rates in patients with intestinal tumor subtype [20], 32]. The intestinal subtype is the most common subtype at the location of the esophagus and the gastroesophageal junction with a prevalence of 72 % here [33]. This means that in AC after neoadjuvant chemotherapy with FLOT, an estimated quota of 20–25 % of all neoadjuvant-treated patients receive surgery in the presence of a locoregional pathologic complete remission.

Comparable to the mentioned pathological complete remission rates after neoadjuvant FLOT chemotherapy, similar results are reported for neoadjuvant chemoradiotherapy.

The CROSS trial described a pathological complete remission rate after neoadjuvant chemoradiation of 23 % in AC patients and even of 49 % in SCC patients [9]. Identification of the complete remission in the mentioned studies was carried out after esophagectomy as pathological complete remission in the surgical specimens. The accuracy of the preoperative identification of patients with complete remission was examined in a recent one-armed feasibility study. The clinical complete remission after neoadjuvant chemoradiation was identified in a specific clinical response evaluation protocol comprising endoscopy with deep biopsies, endoscopic ultrasound, endoscopic ultrasound-guided fine-needle aspiration of suspicious lymph nodes, and 18F-fluorodeoxyglucose positron emission tomography computed tomography (FDGPET-CT) analyzed. The study showed that the highest diagnostic accuracy for the identification of complete response was reached by histology based on endoscopic deep biopsies plus fine needle aspiration of suspicious lymph nodes (10 % false negatives cases/95 % CI 4–23) [34].

A retrospective observational study with 61 patients (SCC n=18, AC n=40, others n=3) with omitted esophagectomy after neoadjuvant chemoradiation in suspicion of complete remission resulted in a 5-year overall survival of 58 %. Overall 13 patients developed local recurrence during follow-up. Twelve of these 13 patients were subsequently treated by salvage esophagectomy [35]. In a subsequent matched-case analysis with 36 patients from this surveillance cohort compared to 36 matched patients undergoing neoadjuvant chemoradiation with subsequent direct esophagectomy, the overall survival was nearly equal in both groups (58 vs. 51 months, p=0.28). The distant metastasis rate was also comparable in both groups (surveillance: 31 % vs. standard surgery: 28 %) [36].

The topic is currently under investigation by three prospectively randomized trials in Europe (NTR 6803, NCT02551458, DRKS00022801) [37]. However, these concepts still have no general justification for any regular clinical application outside of studies.

## Conclusions

Locally advanced SCC of the middle and lower esophagus should be treated with neoadjuvant chemoradiation and subsequent surgery. In severe comorbid patients, definitive chemoradiation is a valuable alternative for curative treatment. Both unimodal esophagectomy and chemoradiation alternatives have inferior oncological outcomes in locally advanced esophageal cancer and should only be used when relevant comorbidities complicate multimodal treatment. Locally advanced esophageal and junctional AC should be treated by neoadjuvant chemoradiation plus esophagectomy or perioperative chemotherapy plus esophagectomy. Up to date, the FLOT protocol has the best evidence for perioperative chemotherapy. In case of a persisting tumor in the operative specimen after neoadjuvant chemoradiation and esophagectomy, the PD-1 inhibitor nivolumab should be given in an adjuvant setting in AC and SCC. The addition of immunotherapeutics in the perioperative setting is supposed to improve the oncological results in the future and is expected to be a part of future treatment standards. Finally, organ-preserving treatment algorithms for patients with clinical complete remission after neoadjuvant treatment are currently tested in clinical trials and may also be a part of future neoadjuvant treatment protocols.
